# Age-friendly design framework for information animations: a mixed-method research validation

**DOI:** 10.3389/fpsyg.2025.1557924

**Published:** 2025-03-07

**Authors:** Xuan Yang, Feng Gan

**Affiliations:** ^1^Guangdong Polytechnic Normal University, Guangzhou, China; ^2^Southeast University, Nanjing, China

**Keywords:** information animation, age-friendly design, perceived ease of use, perceptual experience, information complexity, audiovisual integration

## Abstract

With the rapid aging of the global population, challenges in dynamic visual information processing among older adults have gained increasing attention. Drawing on cognitive aging theory and multimedia cognitive load theory, this study investigated a strategic framework for age-friendly animated information, highlighting the multidimensional nature of dynamic visual cognition in older adults. Using a mixed-methods approach—including literature reviews, interviews, case analyses, and questionnaire surveys—the study examined the effects of perceptual experience (PE), information complexity (IC), and degree of audiovisual integration (AVID) on perceived ease of use (PEOU). The results revealed that PE and AVID significantly and positively influenced PEOU, whereas IC had a significant negative effect. Furthermore, the interaction of multiple independent variables introduces potential interference and complex relationships. Based on these findings, the study proposes a strategic framework for age-friendly animated information, emphasizing the reduction of IC, enhancement of PE, and optimization of AVID to improve information reception among older adults. This framework offers both theoretical guidance and practical support for the development of age-friendly dynamic visual media.

## 1 Introduction

### 1.1 Research background

The aging of the global population has led to increasing attention on the needs and preferences of older adults. In the context of information reception, public service announcements and health-related knowledge for seniors are now predominantly delivered through digital media platforms and mobile communication devices. Consequently, numerous design guidelines (Johnson and Finn, 2017; Falzarano and Czaja, 2024) and solutions (Xu et al., 2020; Wang and Liu, 2021; Jin et al., 2022; Zhu, 2023; Zhou et al., 2024) have focused on interactive interfaces to address the cognitive needs of older adults. However, in a comprehensive review of digital interface design for older adults, only 18% of studies addressed dynamic content considerations. These strategies primarily focus on static visual media design. Similarly, Mayer (2005) review of educational technology research revealed that less than 5% of studies focused on dynamic media design for older learners. The gap is even more pronounced in practical applications. Our survey of educational content across major platforms (YouTube and TED-Ed) found a significant shortage of age-friendly dynamic content. Educational animations specifically designed for older adults comprised less than 2% of educational content. Most existing content failed to incorporate evidence-based design principles for older learners. Few examples demonstrated systematic consideration of age-related cognitive changes.

Age-friendly design strategies for dynamic audiovisual cognition remain underexplored due to two interconnected factors: the cognitive capabilities of older adults and the representational characteristics of dynamic visual media. First, age-related cognitive decline poses significant challenges, including perceptual decay (Baltes and Lindenberger, 1997), diminished attentional inhibition (Brewster et al., 2022), reduced information processing speed (Salthouse et al., 1996), and limited cognitive resources (Schneider and Pichora-Fuller, 2000). These factors collectively affect sensory sensitivity, information processing, memory, and intellectual functioning (Stuart-Hamilton, 2012; Salthouse, 2016), leading to a marked reduction in cognitive efficiency during dynamic audiovisual experiences. Consequently, older adults often lack the cognitive capacity to effectively process dynamic visual information at the intended level. Second, the inherent representational characteristics of dynamic visual media—such as changes in form, position, and outcomes—generate substantial cognitive load (Yang, 2017). The linear temporal progression of dynamic visual content, where new information continuously replaces previous content, places increased demands on cognitive processing and working memory. These challenges are further amplified when dynamic visual media is presented to older audiences.

Paradoxically, human cognition naturally gravitates toward narrative modes that emphasize coherence and linearity (Prince, 2012), which align well with the representational characteristics of dynamic visual media. This is particularly true for procedural information, where the linear structure of dynamic visual narratives closely matches audience cognitive models. Studies have shown that information is most effectively learned when presented in structured and continuous formats (Mayer, 2002). These findings establish the foundational value of age-friendly design research in information animation.

Information animation is a multimodal medium that integrates graphics, images, dynamic visuals, text, and sound. Studies have demonstrated that information animation, as a form of information visualization, offers distinct cognitive advantages helping older audiences achieve their cognitive goals (De Dieuleveult et al., 2017). These advantages include activating sensory compensation mechanisms through audiovisual redundancy (Mahoney et al., 2012), enabling systematic information comprehension via multimodal narrative design, facilitating directional and causal representation of information, reducing cognitive effort for the audience, and stimulating cognitive interest through dynamic visual presentations (Norman, 2019).

For elderly audiences, information animation offers significant communicative advantages by activating sensory compensation, integrating multimodal narratives, providing structured and targeted information representations, and delivering dynamic visual stimulation, highlighting the need for age-friendly design research.

Studies have demonstrated that information animation is an effective medium for lifelong learning (Meyer et al., 2010). As the number of independently living older adults continues to grow, digitally delivered information animation can offer valuable self-care and life guidance, potentially improving their quality of life. Furthermore, dynamic visual narratives are preferred by elderly audiences, who are highly receptive to such narratives (Shir and Asadollahi, 2014; Fang et al., 2019). Consequently, information animation has become a frequently accessed and willingly engaged-in information format for older adults.

Notably, information animation design typically prioritizes key information comprehension over comprehensive detail retention—an approach well-suited to the cognitive capabilities of older adults (Stuart-Hamilton, 2012). Despite challenges such as non-reviewable content and potential dynamic interference, information animation retains substantial communicative value for elderly audiences.

The development of age-friendly design strategies for information animation is a crucial research priority, with the potential to enhance information communication for the growing aging population worldwide.

### 1.2 Research objective

Although information disseminators (Preim and Meuschke, 2020; Sá et al., 2020; Stoll et al., 2021; Chen et al., 2023) have actively explored and confirmed the value of using information animation to communicate health knowledge to elderly audiences, a comprehensive framework for age-friendly design remains unavailable.

In cognitive science, extensive research has focused on cognitive load optimization strategies for information animation (Mayer and Moreno, 2002, 2003; Ploetzner and Lowe, 2012). However, specialized approaches tailored to the cognitive capabilities of older adults remain notably scarce. Although these design strategies are partially applicable to age-friendly design objectives and have been implemented in practice, showing some reference value, they do not comprehensively adapt to elderly persons’ needs. Practice-based studies (Panadisi, 2023) and empirical research (Shi, 2024) have proposed strategies informed by the preferences of the elderly for dynamic visual cognition, including short-duration, simplified information, visual clarity, visual stability, controlled pacing, soft and neutral colors, and audiovisual integration. Despite these contributions, such studies lack theoretical coherence and fail to comprehensively address core age-friendly design strategies. As a result, current design guidelines often lack systematic substantiation and a robust theoretical foundation.

This study employed a mixed-methods approach to identify fundamental strategies and frameworks for age-friendly information animation design, while also examining whether there are interaction effects among relevant factors during strategy implementation.

Our theoretical exploration of an age-friendly design strategy framework for information animation will integrate cognitive aging theory (Salthouse, 2016) and multimedia dynamic visual cognitive load theory (Mayer and Moreno, 2002, 2003). This study adopts a “theory-empirical-theory” scientific validation approach, combining theoretical insights and empirical analysis.

We conducted a comprehensive literature review across relevant disciplines, and in-depth interviews with elderly audiences and visual designers, to identify key factors that enhance cognitive experiences for older adults. This approach addresses the limitations of existing research, which often relies on single-method approaches. Based on the identified factors, we defined predetermined variables and conducted case-based questionnaire surveys to gather sample data, demonstrating the relationships among these variables. The findings were used to theoretically construct an age-friendly design strategy framework for information animation, highlighting the multidimensional complexity of dynamic visual information cognition in older adults. This methodological design effectively addresses the limitations of prior studies, thereby enhancing the credibility of the proposed age-friendly design strategies.

By integrating cognitive science and visual design theories, this study systematically developed age-friendly design strategies to enhance older adults’ cognitive experiences and information comprehension based on empirical findings from interviews and questionnaires. Strategies such as reducing information complexity (IC), enhancing perceptual experiences (PEs), and optimizing the degree of audiovisual integration (AVID) are emphasized. These efforts culminate in the establishment of a foundational framework for age-friendly design guidelines for dynamic visual information.

## 2 Related works

### 2.1 Cognitive aging theory

The decline in cognitive abilities among older adults often begins with diminished information perception, primarily affecting visual and auditory faculties. Visual abilities tend to decline earlier and more significantly, whereas hearing loss typically occurs at a later stage. However, auditory compensation for visual impairments can effectively enhance cognitive experiences (Stuart-Hamilton, 2012).

From the perspective of perceptual function and working memory resource allocation, cognitive aging not only hinders the cognitive processing system’s ability to acquire sufficient information but also leads older adults to expend limited cognitive resources to process new information (Schneider and Pichora-Fuller, 2000). As a result, perceptual and cognitive processing channels compete for these limited resources, leading to reduced cognitive efficiency (Hasher and Zacks, 1988). During audiovisual compensation, the integration of information further strains cognitive resources, making it challenging to manage even simple cognitive tasks effectively (Stuart-Hamilton, 2012). Similarly, complex attentional behaviors and working memory demands in controlled processing exhibit similar competitive relationships (Wang et al., 2017). This conflict among perception, information processing, attention, and memory resource allocation generates varying levels of cognitive load, contributing significantly to the decline in attentional inhibition among older adults.

Cognitive aging theory comprises a range of frameworks that summarize changes in cognitive capabilities among older adults, particularly in attention, information processing, and memory. Key theories include perceptual decline theory (Baltes and Lindenberger, 1997), which describes the incomplete or inaccurate acquisition of sensory information; inhibition function theory (Hasher and Zacks, 1988), highlighting challenges in suppressing irrelevant information and focusing on target information; processing speed theory (Salthouse et al., 1996), characterized by slower information processing and extended reaction times; resource limitation theory (Hasher and Zacks, 1988), which emphasizes reduced thresholds for attention, working memory capacity, and resource utilization in information processing; and the sensory compensation mechanism (Freiherr et al., 2013; De Dieuleveult et al., 2017), describing the benefits of multimodal information integration and added cognitive load generation. These theories collectively form a theoretical foundation for understanding older adults’ cognitive needs and preferences in information animation design.

Perceptual decline, inhibition function, and processing speed theories collectively highlight factors influencing the perceptual stimuli and experiences of older adults engaging with dynamic visual information. In particular, inhibition function, processing speed, and resource limitation of cognitive aging influence elderly audiences’ evaluations of animation information and visual complexity (Tuch et al., 2012). Furthermore, perceptual decline, resource limitation theories, and the sensory compensation mechanism influence older adults’ judgments regarding AVID in information animation.

These five cognitive aging theories highlight distinct factors influencing cognitive load in the perception of information animation by older adults, forming a foundational basis for measuring age-friendly information animation dimensions. Table 1 presents the measurement dimensions impacting cognitive evaluation.

**Table 1:** Cognitive aging theory and evaluation of information perception.

Cognitive aging theory	Dimensions affecting the cognitive evaluation	Methods for reducing cognitive load	Cognitive evaluation
Perceptual decline theory Inhibition function theory Processing speed theory	PE Stimulus perception Perceptual experience	Controlling color stimulation Enhancing keyword visualization Visual clarity Controlling image speed Selecting speech rate	Cognitive load ↓ Easy-to-use perception
Perceptual decline theory Inhibition function theory Processing speed theory Resource limitation theory Sensory compensation mechanism	IC Intrinsic IC Extraneous IC Germane IC	Reducing visual complexity Designing text information description Removing visually interesting but irrelevant graphics or symbols Reducing information complexity Offering prior knowledge to audience Providing visual cues Visually segmenting information	
Perceptual decline theory Resource limitation theory Sensory compensation mechanism	AVID Compensation effect Information integration effect	Synchronized audiovisual channels	

### 2.2 Cognitive load theory

Animation representation demands substantial cognitive resource allocation, posing particular challenges for older adults. Cognitive load issues are common in the dynamic audiovisual processing of elderly audiences (Stuart-Hamilton, 2012), often hindering information reception and reducing working memory efficiency. Therefore, addressing cognitive load is a crucial consideration in age-friendly design.

Cognitive load is categorized into three types (Sweller, 1988). Intrinsic cognitive load arises from the inherent complexity of a task. This load is task-specific and cannot be eliminated, although it can be managed using content segmentation and simplification. Extraneous cognitive load is generated by non-essential elements within the task, such as irrelevant information, overly complex designs, redundant text and images, and poor audiovisual integration (AVI) which increases the cognitive burden. Germane cognitive load is associated with cognitive processes, reflecting the resources dedicated to information retrieval and processing, constructing new mental models, and forming long-term memory.

The three types of cognitive load encompass variables such as task complexity, information content complexity, presentation methods, AVI, and prior knowledge, which influence how older adults process animated information. Theoretically, these factors connect mental models and visual communication design with cognitive load. Practically, they relate to elderly PEs (WHO, 2015), complexity perception (Lazard and Mackert, 2014), and the effects of multimodal information integration (Van Gerven et al., 2006), all of which collectively impact cognitive load intensity. In design practice, minimizing extraneous cognitive load, managing intrinsic cognitive load, and enhancing germane cognitive load (e.g., through step-by-step demonstrations) offer a clear pathway to improving perceived ease of use (PEOU) in information animation.

Cognitive load processing involves two key aspects: identifying cognitive load issues and optimizing them through design (Sweller, 1988). The evaluation of cognitive load serves as a crucial indicator of ease of use in information animation (Mayer, 2002).

Mayer and Moreno (2002, 2003) proposed several methods to reduce cognitive load in information animation, such as synchronizing audiovisual channels, visually segmenting information, removing visually engaging but irrelevant graphics or symbols, offering prior knowledge to audiences, and providing hints. These strategies have been validated by numerous studies (Schmidt-Weigand et al., 2010; Spanjers et al., 2010; Hatsidimitris and Kalyuga, 2013; Canham and Hegarty, 2010; Jarodzka et al., 2010; Barnes, 2017; Boucheix et al., 2013). Additionally, Ploetzner and Lowe (2012) synthesized strategies from 44 studies, including providing cues, controlling image speed, adjusting speech rate, designing clear text descriptions, reducing IC, and enhancing spatial continuity. Several components of Mayer and Moreno’s summary were further identified.

Although cognitive science research on cognitive load optimization strategies does not address the cognitive abilities of older adults, these strategies serve as valuable references for universal information animation design and are considered equally applicable to age-friendly contexts. Building on this foundation, Panadisi (2023) implemented age-friendly information animation designs, incorporating strategies such as reducing information and visual complexity, controlling color stimulation, enhancing keyword visualization, and providing visual cues. Similarly, Shi (2024) collected data from elderly audiences on animation speed, visual clarity, information volume, comprehension, viewing frequency, and preferences, subsequently proposing age-friendly design principles that emphasize visual clarity and information simplification.

## 3 Research dimension

Drawing on research into elderly cognitive abilities and dynamic visual cognitive load, and synthesizing findings from existing age-friendly information animation design cases and empirical studies, this study tentatively identified three primary factors influencing the effectiveness of age-friendly information animation design: PE, IC, and AVID (Table 1). The study hypothesizes that these factors significantly impact the cognitive load experienced by elderly audiences. Furthermore, the evaluation of cognitive load is proposed as a crucial indicator for assessing the ease of use of information animation (Mayer, 2002).

### 3.1 PEOU

PEOU, introduced in the technology acceptance model (Davis, 1989), is a key determinant of user acceptance and adoption of technology. Users are more likely to adopt and use a system or product when they perceive it as easy to use.

PEOU serves as a key outcome indicator in evaluating whether age-friendly information animations are accepted by elderly audiences, given its direct relationship with cognitive load. Reducing cognitive load has been shown to enhance PEOU, a connection supported by the overlap between cognitive load evaluation metrics (Paas et al., 2016) and PEOU measurement indicators (Davis, 1989).

Audiences tend to perceive an information product or learning material as more user-friendly when it imposes low cognitive load. Conversely, high cognitive load reduces PEOU. When a visual information product is overly complex and demands significant cognitive resources to process, audiences may struggle to comprehend the content, ultimately lowering their intention to engage with it.

Effectively managing and reducing cognitive load across intrinsic, extraneous, and germane dimensions is theoretically linked to enhancing the PEOU of information products. Building on Davis’s (1989) PEOU scale, this research utilizes questionnaire surveys to assess elderly audiences’ challenges in maintaining attention, understanding content, and acquiring knowledge from animated information as key indicators of PEOU.

### 3.2 PE

Fisk et al. (2009) and Stuart-Hamilton (2012) demonstrated the decline in visual sensitivity and auditory discrimination with age, emphasizing the need for age-friendly cognitive media designs that prioritize enhancing perceptual stimulation. However, Norman and Bobrow’s perceptual load theory (Norman and Bobrow, 1975) suggests that excessive perceptual stimulation can lead to physiological discomfort in older adults and deplete their limited cognitive resources, thereby impairing information processing efficiency. McLaughlin and Pak (2020) highlight the importance of tailoring the intensity of visual information presentation to the tolerance levels of elderly audiences, noting that overly intense or insufficient stimuli can hinder information acquisition. Shneiderman and Plaisant (2014) argue that animation design should balance effective information transmission and an enjoyable viewing experience, emphasizing the need to prioritize audience perceptual comfort. The World Health Organization (WHO, 2015) identifies perceptual accessibility as a crucial evaluation criterion in age-friendly design guidelines. Additionally, Schrepp et al. (2014) proposed a PE assessment framework that evaluates factors such as clarity, perceptual efficiency, and attractiveness.

Building on these insights, this research identifies PE as a critical indicator for evaluating the PEOU of information animations among elderly audiences. The primary observational dimensions include visual perceptual clarity, perceptual comfort, and adaptability to dynamic rhythm.

### 3.3 IC

Stuart-Hamilton (2012) and Salthouse (2016) highlight that information processing capabilities gradually decline with age, manifesting as reduced working memory abilities, slower information processing speeds, and challenges in attention allocation. Cognitive load theory and resource limitation theories of aging emphasize the importance of reducing intrinsic IC to enhance cognitive experience and efficiency for older adults (Paas and van Merriënboer, 2020). Similarly, visual complexity (extraneous IC) significantly affects audience perceptions of information ease of use through their overall impression of the information (Tuch et al., 2012). The cognitive complexity of processing information animation (germane IC) involves allocating cognitive resources for querying, processing, constructing mental models, and forming long-term memory. Mayer and Moreno (2003) identified inconsistencies between design and audience mental models as a key factor in increasing cognitive load. Lazard and Mackert (2014) further demonstrated that complexity factors influenced by design should be treated as variables in information dissemination and prioritized in design strategies.

This research identified IC as the core indicator for evaluating elderly audiences’ PEOU of information animations. The observed content aligns directly with the three types of cognitive load. By referencing the information complexity scales of Mayer (2002) and visual complexity scales of Tuch et al. (2009), while considering the cognitive characteristics of older adults and the representational features of information animation, this study incorporates factors such as information volume, sustained viewing tolerance, narrative structure comprehension, visual perception complexity, and semantic understanding into the assessment of IC.

### 3.4 AVID

AVI is recognized as a key factor in enhancing elderly audiences’ PEOU in information animations, primarily through sensory compensation mechanisms and the functional advantages of audiovisual media. The human brain’s “multimodal” processing capabilities allow it to integrate information from different sensory channels, thereby improving cognitive efficiency (Freiherr et al., 2013; De Dieuleveult et al., 2017). Given their declining sensory abilities, older adults often rely on sensory compensation mechanisms (Salthouse, 2016). However, cognitive load theory and resource limitation theory indicate that multimodal information processing in older adults demands additional cognitive resources, potentially increasing the complexity of information processing. This theoretically suggests a possible inverse relationship between multimodal integration effects and the perceived complexity of information.

Talsma et al. (2007) emphasized that the effectiveness of multisensory integration relies on the comprehensive engagement of multisensory stimuli. For older adults with diminished vision or hearing capabilities, this perspective underscores that sensory compensation effects should be a central focus in the age-friendly design of information animations.

Consequently, the primary design objective for integrating information in elderly multimedia cognitive environments is to activate sensory compensation mechanisms. This involves simplifying AVI steps, balancing the proportion of visual and auditory information processing, and reducing the overall processing difficulty.

This research evaluates AVID by emphasizing auditory compensation for visual perception and examining the relationships within AVI across three critical cognitive stages: attention, information processing, and memory. The study is tailored to the cognitive characteristics of elderly audiences and the representational features of information animation.

### 3.5 Hypothesis formulation

The study proposes PE, IC, and AVID as independent variables hypothesized to significantly influence elderly audiences’ PEOU evaluation of animated information. The hypotheses are as follows:

*H1*: PE significantly and positively impacts PEOU.

*H2*: IC significantly and negatively impacts PEOU.

*H3*: AVID significantly and positively impacts PEOU.

The three hypotheses collectively establish a comprehensive theoretical model, demonstrating the multidimensional factors that influence elderly audiences’ PEOU of information animations. This model provides a strategic framework for age-friendly information animation design. H1–3 illustrate that PEOU emerges from the combined effect of multiple constructs, each with distinct influences: an increase in IC is associated with a decrease in PEOU, whereas improvements in PE or AVID correlate with an increase in PEOU.

## 4 Methods and materials

### 4.1 Research strategy

This research adopts a mixed-methods approach, employing an iterative “theory–empirical–theory” process to thoroughly investigate and refine design strategies that enhance the reception and cognitive processing of dynamic visual information by older adults. This approach aims to establish a comprehensive framework for age-friendly design strategies.

The research followed these specific steps:

Step 1: theoretical analysis × in-depth interviews

Key factors enhancing cognitive experiences in information animations for older adults were identified, confirming the relevant variables.

Step 2: case selection × questionnaire survey

Animation cases were carefully selected, and sample data were collected via questionnaire surveys to validate the proposed hypotheses and explore the interactional relationships between variables.

Step 3: theoretical construction

The verified impact factors were transformed into a theoretical framework. This framework clarifies age-friendly design strategies for information animations and reveals the multidimensional complexity of cognitive processing of dynamic visual information for older adults.

The selection of variables and construction of hypotheses were grounded in rigorous cognitive science theories, while also addressing the practical cognitive needs of elderly audiences. This approach exemplifies a seamless integration of theoretical research and empirical exploration.

### 4.2 In-depth interviews

This study explored cognitive theories related to aging, information animation cognitive load theories, and relevant empirical literature. Our preliminary analysis identified three potential factors—PE, IC, and AVID—as significant contributors to the cognitive load experienced by older adults, potentially influencing their PEOU evaluations. To confirm the relevance and impact of these factors, we conducted in-depth interviews with elderly participants and industry professionals, including visual designers.

#### 4.2.1 Interview design

Interviews with elderly participants aimed to gain a deeper understanding of the challenges they face with existing information animations, as well as their specific needs and preferences. Expert interviews were conducted to validate and expand on the cognitive challenges and needs identified in the interviews with elderly participants. Additionally, these interviews gathered insights from designers on practical experience with age-friendly information animation design and specific recommendations for improving such designs. Each group consisted of five participants. The expert interviews, conducted after those with elderly participants, were completed in October 2024. All interviews followed a semi-structured, one-on-one format.

Elderly participants were selected from three culturally distinct regions in China: South, Southwest, and Southeast. Participant selection carefully balanced gender, age, and educational backgrounds. Women were slightly overrepresented in the higher age group, as they typically exhibit better hearing and memory than men of the same age (Stuart-Hamilton, 2012), whereas men were prioritized in the lower age group to better represent a range of cognitive–physiological conditions. Recognizing that educational background significantly influences cognitive abilities, interviews in the higher age group focused on participants with higher education levels, whereas those in the lower age group emphasized individuals with lower educational attainment. This approach aimed to comprehensively capture the cognitive needs and preferences of the elderly population. All participants were screened to ensure the absence of significant vision or hearing impairments. Prior to the formal interviews, participants viewed a popular science animation on the topic of heatstroke to stimulate recall and provide a foundation for their responses regarding information animations.

The expert interviews involved five professionals: two media designers with experience in age-friendly design, one information animation designer, one information visualization designer, and one expert in media design and communication. All participants had over 7 years of professional experience.

The overall objective of the interviews was to provide empirical support for the theoretically proposed constructs and their hypothesized influencing pathways, further clarifying the predefined variables affecting perceptions of ease of use. The interview records were carefully annotated and coded to identify valuable information and key insights, highlighting logical connections between different themes and forming a proof chain.

#### 4.2.2 Interview information

##### 4.2.2.1 Elderly audience interview information

The interview primarily focused on the elderly participants’ viewing experiences, cognitive needs, and preferences. All participants provided feedback based on their impressions after viewing the animation. Common observations were that the “animation duration was slightly long” and that “a slower playback speed would be more effective.” Participants suggested that dividing the animation into shorter segments with clear themes would better sustain attention and enhance memory retention. These insights indicate that shorter durations and slower playback speeds are more aligned with the preferences of elderly viewers.

Theoretically, animation duration and playback speed influence factors such as information volume, attention shifts, and cognitive load, all of which affect the assessment of IC. During the interviews, two participants expressed that they “prefer simple visuals and dislike complex information and graphics.” These preferences highlight the elderly audience’s heightened sensitivity to IC, suggesting its significant impact on PEOU and overall cognitive experience.

Regarding PE, Participant A noted that “the animation visuals seem chaotic, though this did not hinder understanding,” whereas Participant C remarked that “unnecessary patterns in the animation are superfluous, making the image complex.” Participant A’s perception of visual disorder was mainly attributed to the excessive graphic elements clustered in static frames, a point also noted by Participant C, who suggested their removal. Additionally, frequent graphic movements were perceived as detracting from the viewing experience, with Participant A describing it as “messy.” These visual distractions diverted attention from key information, leading to an overall impression of “high visual complexity.”

Regarding audiovisual information integration, Participant A stated that “information acquisition through auditory channels is effective,” and Participant E agreed, noting that “sound helps with information comprehension.” Participant C felt that “audiovisual combination enhances understanding and memory,” and Participant D believed that “text–image combination better supported understanding.” These responses collectively highlight the elderly audience’s preference for multimodal information.

##### 4.2.2.2 Expert interview information

The analysis of expert interviews focused on three potential influencing factors from the theoretical framework: PE, IC, and AVID.

Regarding PE, Expert A pointed out that excessive visual background information could distract the audience and suggested focusing on auditory information design, considering the prevalence of late-stage hearing loss in the elderly population. Expert B emphasized the importance of font contrast in age-friendly design, noting that font selection significantly affects visual recognition. He also recommended limiting the number of visual stimuli or objects in single frames, ensuring they are sufficiently stimulating without using overly vibrant or flashing colors that could cause physiological discomfort for elderly viewers.

Regarding IC, Experts A, B, C, and E unanimously emphasized the need to reduce complexity in three key areas: information content, visual arrangement, and narrative structure. The goal was not only to prevent cognitive resource competition between attention and information processing but also to mitigate resource conflicts within each stage of processing. Expert A suggested that age-friendly information animations should generally feature simple scenes, a central visual focus, high contrast, and linear narratives to minimize cognitive load and conflict. Expert B emphasized the importance of avoiding information overload and excessive content difficulty, recommending that content be categorized into no more than four categories and that one information unit be presented at a time to ensure efficient processing.

Regarding AVID design, Expert A highlighted the importance of auditory information, particularly given the need for hearing-impaired elderly audiences to rely solely on visual means for comprehension. She recommended using visual language to clearly introduce the content background while balancing clarity with appropriate complexity. Expert E emphasized that maintaining information consistency through redundancy strategies helps capture the attention of elderly viewers with shorter attention spans and supports more effective information processing.

In discussing age-friendly information animation design strategies, all five experts recommended enhancing and controlling the intensity of visual stimulation, reducing information content and narrative complexity, and creating concise, intuitive visuals. These suggestions align directly with the key strategies identified in the theoretical exploration: enhancing visual sensory stimulation and perceptual experience while simultaneously reducing IC to minimize cognitive resource allocation.

#### 4.2.3 Interview results

The analysis of elderly audience interviews provided key insights: elderly viewers prioritize IC, which directly influences their ease of use evaluations and cognitive experience. They also believe that effective integration of visual, auditory, graphical, and textual elements enhances understanding and memory. Keywords frequently mentioned during the interviews—such as animation duration, visual speed, complexity-forming elements, sound, speech rate, and audiovisual synchronization—can serve as the foundation for adapting a questionnaire scale, thereby laying the groundwork for questionnaire design.

The analysis of expert interviews yielded key insights: the proposed age-friendly information animation design strategy framework aligns closely with the recommendations and schemes provided by design experts. Key aspects addressing PE, IC control, and audiovisual element processing directly correspond to the study’s three predetermined independent variables, thereby refining the factors for questionnaire scale composition and clarifying the survey’s objectives.

Based on cognitive aging theory and interview analysis, four core variables for age-friendly information animation design can be preliminarily identified: PE, IC, AVID, and PEOU. PE, a key variable reflecting elderly cognitive acceptance, captures perceptual characteristics of information animations through the dimensions of visual clarity and comfort. IC addresses the limited cognitive resources and working memory capacity of older adults, illustrating the impact of complexity on cognitive processing through indicators such as information volume, narrative structure, and semantic understanding. AVID pertains to sensory compensation mechanisms in multimodal information processing, highlighting the elderly audience’s need for audiovisual coordination across attention, comprehension, and memory. PEOU, as the dependent variable, comprehensively evaluates the combined impact of these three dimensions on cognitive load and information reception, serving as a crucial indicator of age-friendly design effectiveness.

Through cross-analysis of interview data and cognitive theories, the study’s three core hypotheses—(H2) IC significantly negatively impacts PEOU, and (H1 and H3) PE and AVID significantly positively impact PEOU—can be further validated through questionnaire survey data analysis. This process will establish a framework for age-friendly information animation design strategies.

### 4.3 Questionnaire survey

#### 4.3.1 Sample collection

A small-scale pre-test of the questionnaire survey targeting elderly audiences was conducted in October 2024. The formal survey took place in November, with online and paper questionnaires distributed to Chinese individuals aged 60 years and above via social networks, both directly and indirectly. A total of 90 valid responses were collected from participants across five regions of China: Southwest, South, Central, North, and Northeast.

The age distribution of healthy respondents was as follows: 60–69 years (61.1%), 70–79 years (32.2%), and ≥ 80 years (6.7%). The gender composition was 33.3% male and 66.7% female. Regarding educational background, 41.1% of the respondents had completed junior high school or below, 41.1% had attended high school, and 17.8% had obtained a college degree or higher. In terms of vision, 64.4% of the respondents had normal vision, whereas 35.6% had mild vision issues, with no respondents reporting moderate or severe vision problems. Concerning hearing, 68.9% of the respondents had normal hearing, 25.6% had mild hearing issues, and 5.5% had moderate hearing problems, with no severe hearing impairments. The survey sample effectively represented the target population, offering a diverse range of characteristics.

Participants were instructed to watch the case animation before completing the questionnaire.

#### 4.3.2 Case selection

**Figure 1:**
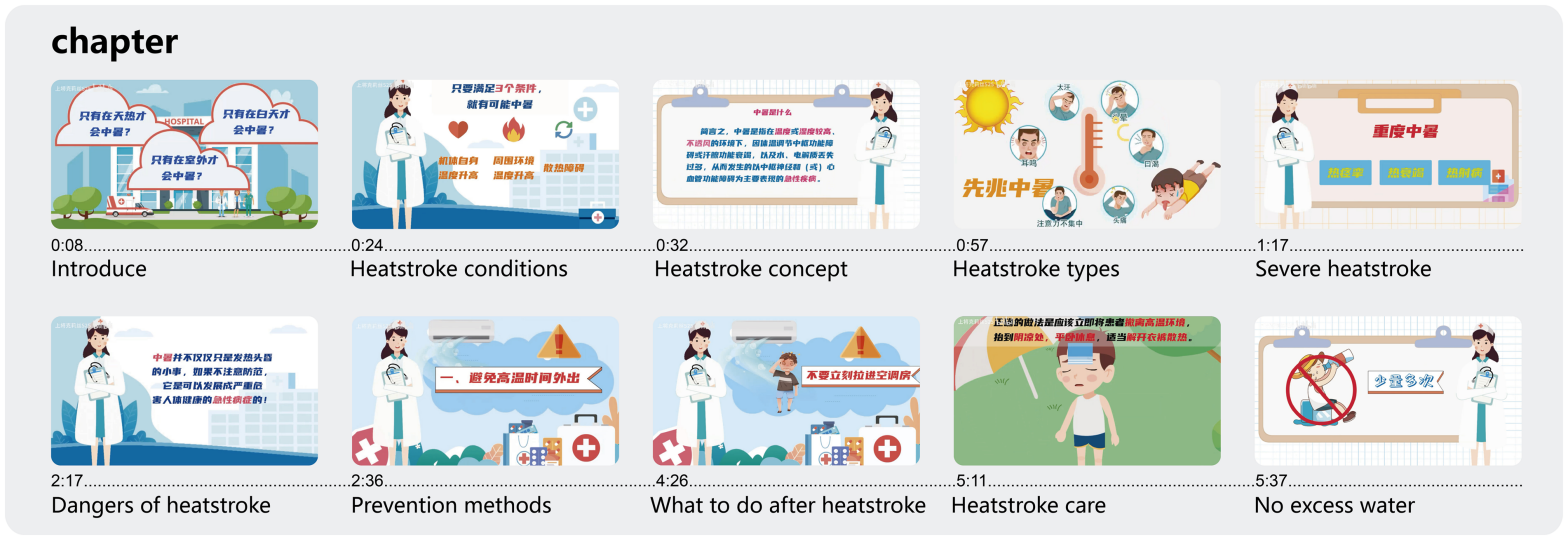
Information animation case—heatstroke. Source: https://www.bilibili.com/video/BV1o1421v72T/?spm_id_from=333.337.search-card.all.click.

The animation used for the questionnaire survey and interviews was a popular scientific animation introducing the topic of heatstroke (Figure 1). With a duration of 5 min and 50 s, the animation is suitable for a general adult audience but may be overwhelming for elderly viewers due to its large information volume and extended viewing time. The information-content complexity is moderate, incorporating some professional knowledge. The visual presentation combines graphics and text, includes design-induced visual interference elements, features inappropriate dynamic visual components, and uses a female voiceover.

Although this case cannot fully represent all types of information animations, it exemplifies a common form of information animation that elderly adults frequently encounter across various information platforms. This animation format fundamentally aligns with elderly cognitive models and represents a type that will be commonly applied in future age-friendly animation design.

##### 4.3.2.1 Theme selection

Public health and medical topics are the preferred information types for elderly audiences, which helped avoid potential survey bias that might arise from participants’ lack of interest in the subject matter. Heatstroke knowledge, in particular, is widely applicable and does not require professional expertise to comprehend. The overall content is easily understandable, making it well-suited to the cognitive processing abilities of elderly individuals.

##### 4.3.2.2 Duration selection

Guo et al. (2014) determined that the optimal length for online learning videos is 6 min or less. Industry analyses similarly indicate that social media platforms such as YouTube, Facebook, and Instagram sustain user attention within a range of 5–7 min. TED-Ed educational videos also follow this model, offering animations within the same time frame. This duration effectively balances the transmission of information with the maintenance of audience focus. Notably, 5–7-min animations are prevalent in online resources and are familiar to elderly audiences, striking a balance between avoiding disengagement due to excessive length and adequately meeting cognitive needs.

##### 4.3.2.3 Narrative structure and information volume

The animation presents information across five segments: heatstroke conditions, definition, developmental stages and symptoms, prevention strategies, and post-heatstroke management. It employs a linear narrative structure tailored to align with the cognitive narrative preferences of elderly audiences. However, the substantial volume of information poses challenges to their attention span, information processing, and memory capabilities. This structure provides an opportunity to identify factors that influence cognitive efficiency and user experience.

##### 4.3.2.4 Information-content complexity

The animation is organized into five clearly defined segments. However, the “developmental stages and symptoms” segment presents complex internal relationships, has high information density, uses extensive medical terminology, and requires professional knowledge. The “post-heatstroke management” segment combines procedural and declarative information, resulting in loose logical connections and greater memory demands. Although the overall complexity of the animation is not inherently high, these factors incrementally increase cognitive load for the audience.

##### 4.3.2.5 Visual complexity

The animation features numerous visuals, including frequently overlapping graphics and cluttered layouts. Blinking visual elements, intended to capture attention, highlight a common design flaw that can be analyzed to explore patterns of audience attention shifts. Additionally, dense text sections and frequent, rapid, and dynamic visual changes further increase the perceived visual complexity, significantly impacting the PEs of elderly viewers.

##### 4.3.2.6 Text–image relationship

The animation’s static visual layouts incorporate dense text arrangements and text–image combinations, providing an opportunity to explore how various dynamic visual layouts influence elderly audiences’ perception of IC, visual search efficiency, and the effectiveness of text–image integration.

##### 4.3.2.7 Audio information

Considering that the high-frequency vocal ranges of female voices exceed those of male voices, this study intentionally selected animations with female voiceovers to examine whether the characteristics of female voices might adversely affect PEs and AVI in elderly participants. Although the narration’s semantic content is straightforward and conversational, the speech rate of 250 characters per min notably surpasses the standard 220 characters per min, further challenging information processing and AVI.

The selected information animation was thoroughly analyzed across several dimensions, including theme, duration, narrative structure, IC, visual complexity, text–image relationships, and sound. By integrating cognitive aging theories, cognitive preferences, dynamic visual cognitive detection indicators, and factors influencing cognitive experiences, this case serves as a representative model for examining research variables and testing hypotheses.

#### 4.3.3 Questionnaire design

The questionnaire was designed to investigate data across four dimensions: elderly audiences’ PEs of information animations, elderly audiences’ assessments of IC, the effects of AVID, and elderly audiences’ evaluations of PEOU. A five-point Likert scale (Likert, 1932) was used for scoring. To minimize comprehension difficulties for elderly respondents, all questions were framed using positive statements and expressed in short sentences. Table 2 presents the dimensions and specific items of the questionnaire.

**Table 2:** Questionnaire design.

Dimensions	Items	Scoring formula
Information complexity (IC) Adapted from Mayer (2002) and Tuch et al. (2009)	IC1 Not many animation visual elements IC2 Screen layout presents information in a simplified manner IC3 All text on the screen can be read IC4 Despite text density, information appears uncomplicated IC5 Narrative structure of animation is straightforward IC6 Animation duration enables sustained attention IC7 Voiceover semantic content is simple and easily comprehensible IC8 Voiceover pace is moderate, allowing cognitive processing ICZ Watching this animation was effortless	Strongly agree −1 Agree −2 Neutral −3 Disagree −4 Strongly disagree −5
Perceptual experiences (PE) Adapted from UEQ (Schrepp et al., 2014)	PE1 Animation colors are not harsh PE2 Animation colors are not chaotic PE3 Animation color differences are distinct, facilitating information differentiation PE4 Animation visual elements are not disorganized PE5 Movement speed of animation elements is not rapid PE6 Moving elements do not create a dizzying visual effect PE7 Graphic flickering does not interfere with attention to other information PE8 Voiceover content is clearly audible PEZ Animation provides a comfortable viewing experience	Strongly agree −5 Agree −4 Neutral −3 Disagree −2 Strongly disagree −1
Degree of audiovisual integration (AVID)	AVID1 Voiceover volume is appropriate AVID2 Audio information effectively aids in comprehending visual content AVID3 Sounds direct attention to specific visual elements AVID4 Audio information enhances visual content retention AVIDZ Audiovisual integration supports cognitive processing	
Perceived ease of use (PEOU) Adapted from PEOU (Davis, 1989)	PEOU1 Maintaining attention on the animation requires no significant effort PEOU2 Animation is clear and easily comprehensible PEOU3 Watching the animation does not demand substantial mental or cognitive resources PEOU4 Animation facilitates effortless knowledge acquisition	

#### 4.3.4 Questionnaire data analysis

##### 4.3.4.1 Basic data analysis

###### 4.3.4.1.1 Descriptive statistics (Table 3)

**Table 3:** Data characteristics, distribution, and multicollinearity.

Indicator	Mean	Median	Standard deviation	VIF	Skewness	Kurtosis	Jarque–Bera test	
							χ^2^	*p*
PE1	4.011	4	0.782	2.574	−0.304	−0.600	2.839	0.242
PE2	3.944	4	0.848	3.963	−0.560	−0.170	4.733	0.094
PE3	3.911	4	0.740	2.928	−0.357	−0.004	1.863	0.394
PE4	4.011	4	0.738	4.972	−0.018	−1.160	5.074	0.079
PE5	3.656	4	0.991	4.094	−0.373	−0.874	4.999	0.082
PE6	3.811	4	0.918	4.566	−0.401	−0.619	3.925	0.141
PE7	3.644	4	0.970	2.720	−0.120	−0.969	3.823	0.148
PE8	3.911	4	0.927	2.603	−0.757	−0.118	8.433	0.015*
PEZ	4.011	4	0.823	3.327	−0.264	−0.915	4.257	0.119
IC1	2.322	2	0.987	4.308	0.438	−0.806	5.355	0.069
IC2	2.056	2	0.947	3.393	0.686	−0.356	7.423	0.024*
IC3	2.489	2	1.118	2.520	0.392	−0.811	4.832	0.089
IC4	2.144	2	0.984	4.001	0.629	−0.251	6.074	0.048*
IC5	2.067	2	0.952	2.694	0.571	−0.585	6.170	0.046*
IC6	2.356	2	1.068	2.883	0.413	−0.867	5.412	0.067
IC7	1.933	2	0.879	4.267	1.129	1.422	24.611	0.000**
IC8	2.244	2	1.109	3.079	0.643	−0.582	7.421	0.025*
ICZ	2.300	2	1.110	3.262	0.667	−0.244	6.785	0.034*
AVID1	4.078	4	0.806	2.002	−0.662	0.084	6.363	0.042*
AVID2	4.144	4	0.754	3.490	−0.566	−0.082	4.715	0.095
AVID3	4.000	4	0.843	2.421	−0.678	0.057	6.675	0.036*
AVID4	3.989	4	0.810	2.722	−0.874	1.414	17.132	0.000**
AVIDZ	4.133	4	0.777	2.578	−0.528	−0.341	4.613	0.100
PEOU1	3.611	4	1.142	3.258	−0.370	−1.151	6.981	0.030*
PEOU2	3.911	4	0.890	4.472	−0.303	−0.820	3.990	0.136
PEOU3	3.811	4	0.942	4.326	−0.422	−0.677	4.455	0.108
PEOU4	4.156	4	0.802	2.598	−0.951	0.840	15.096	0.001**

*p < 0.05, **p < 0.01. *n* = 90.

The survey yielded 90 valid samples, all free of missing or anomalous values, indicating high data quality. A total of 27 indicators across the four predefined variables were analyzed. The mean values ranged from 1.933 to 4.156, with standard deviations between 0.74 and 1.142, reflecting moderate dispersion. The median and mean values were closely aligned, and the standard deviations were relatively small in relation to the means, suggesting a concentrated data distribution. No extreme values or significant skewness were observed.

###### 4.3.4.1.2 Normality test (Table 3)

The Jarque-Bera test was employed to examine the skewness and kurtosis of the data for comprehensive and reliable assessment. The *p*-values indicated that some indicators (e.g., IC7, AVID4, and PEOU4) deviated from the assumption of a normal distribution (*p* < 0.01). However, all skewness and kurtosis values were close to 0 (range: −2 to 2), suggesting a symmetric and concentrated distribution approximating normality (Garson, 2012).

###### 4.3.4.1.3 Variable indicator correlation test

Pearson correlation analysis revealed statistically significant correlations among all observed indicators (p < 0.01, within 99% confidence intervals). The latent variable correlation coefficients were as follows: PE, 0.446–0.791; IC, 0.512–0.789; AVID, 0.458–0.707; and PEOU, 0.394–0.820; these values indicate high internal consistency, yet they do not completely overlap (< 0.85), demonstrating the effectiveness of the measurement.

###### 4.3.4.1.4 Multicollinearity test (Table 3)

Variance inflation factor (VIF) analysis showed that all indicator VIF values were below the empirical threshold of 5, indicating the absence of multicollinearity among the indicators and predefined variables.

##### 4.3.4.2 Measurement model for latent variables

Factor analysis (Table 4) revealed that 74.067% of the cumulative variance was explained after rotation, indicating that the 27 observed indicators provide strong explanatory power for the four predetermined variables and demonstrating the robust information-capturing ability of the measurement model. The Cronbach’s *α* coefficient of 0.625 confirms that the sample’s responses meet the standard for authenticity (with values >0.6 considered acceptable). The overall dataset’s Kaiser–Meyer–Olkin (KMO) value of 0.845 (with KMO values of 0.8–0.9 classified as “very good”) and p-value of 0 demonstrate the data’s reliability and suitability for factor analysis, further confirming the overall construct validity and explanatory power of the model.

**Table 4:** Factor loadings.

	Factor 1	Factor 2	Factor 3	Factor 4
Cumulative variance explained (after rotation)	21.907%	41.344%	58.856%	74.067%
*Cronbach’s α*	0.625			
KMO	0.845			
p	0.000**			

*p < 0.05, **p < 0.01.

The latent independent variables consist of formative indicators: 9 for PE, 9 for IC, and 5 for AVID. The latent dependent variable (PEOU) includes 4 reflective indicators. Further reliability and validity testing of these four latent variables revealed Cronbach’s α coefficients >0.8, KMO values >0.7, factor loading coefficients >0.7, and R^2^ values (communalities) > 0.4, demonstrating the excellent internal consistency and validity of the measurement model (Table 5). All observed indicators effectively reflect the characteristics of the latent variables.

**Table 5:** Reliability and validity of predetermined independent variables.

Indicator	Factor loadings	*R^2^*	α coefficient with item deleted	Cronbach’s α	KMO	*p*
PE1	0.715	0.512	0.933	0.934	0.839	0.000**
PE2	0.836	0.699	0.925			
PE3	0.790	0.624	0.928			
PE4	0.874	0.764	0.923			
PE5	0.847	0.718	0.924			
PE6	0.850	0.723	0.924			
PE7	0.779	0.607	0.929			
PE8	0.795	0.632	0.928			
PEZ	0.834	0.695	0.925			
IC1	0.868	0.754	0.932	0.942	0.908	0.000**
IC2	0.782	0.611	0.938			
IC3	0.814	0.663	0.936			
IC4	0.868	0.754	0.933			
IC5	0.786	0.618	0.938			
IC6	0.815	0.664	0.936			
IC7	0.863	0.744	0.934			
IC8	0.828	0.685	0.936			
ICZ	0.840	0.705	0.934			
AVID1	0.755	0.571	0.876	0.880	0.745	0.000**
AVID2	0.885	0.784	0.835			
AVID3	0.809	0.654	0.860			
AVID4	0.840	0.705	0.848			
AVIDZ	0.829	0.688	0.854			

*p < 0.05, **p < 0.01.

Normality testing, multicollinearity assessment, correlation analysis, and validity evaluation confirmed the scientific rigor and reliability of the measurement model. The results indicate good fit and high quality of the measurement model, providing a solid foundation for subsequent studies.

##### 4.3.4.3 Latent variable relationships

**Figure 2:**
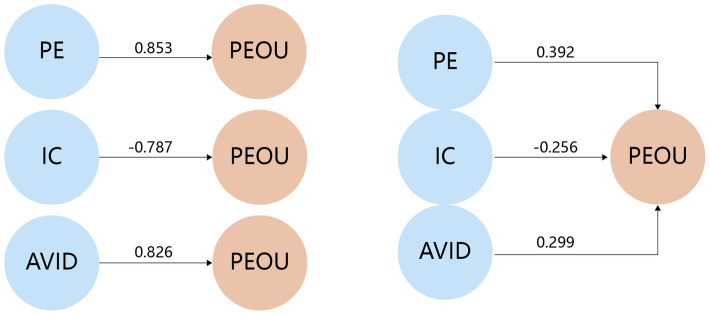
Detection model. **(Left)** Independent factor detection model; **(Right)** Joint effect model.

The path relationships between the latent variables are shown in an “independent factor detection environment” (left-side model in Figure 2). Path analysis (data from the left side of Table 3) revealed that PE had a strong positive impact on PEOU (path coefficient = 0.853, *p* = 0, *f* ^2^ = 2.682), whereas IC had a significant negative influence on PEOU (path coefficient = −0.787, *p* = 0, *f* ^2^ = 1.629). AVID also had a significant positive effect on PEOU (path coefficient = 0.826, *p* = 0, *f* ^2^ = 2.154). These findings suggest that for elderly audiences, higher PE, lower IC, and better AVID are associated with stronger PEOU (Table 6).

**Table 6:** Influences and effects.

Path	Independent factor detection model			Joint effect model		
	Path coefficients	*f^2^*	*p*	Path coefficients	*f^2^*	*p*
PE ➔ PEOU	0.853	2.682	0.000**	0.392	0.124	0.003**
IC ➔ PEOU	−0.787	1.629	0.000**	−0.256	0.111	0.045*
AVID ➔ PEOU	0.826	2.154	0.000**	0.299	0.100	0.012*

*p < 0.05, **p < 0.01.

Further analysis using Pearson correlation allowed the construction of a correlation matrix of the variables (Table 7). This analysis revealed significant correlations between PE, IC, AVID, and PEOU, verified the discriminant validity of the variables, and explored potential interactions between them.

**Table 7:** Correlation coefficient matrix of the variables.

	PE	IC	AVID	PEOU
PE	1			
IC	−0.790	1		
AVID	0.866	−0.692	1	
PEOU	0.854	−0.773	0.816	1

Inter-variable correlations: The matrix based on the path coefficient analysis revealed significant associations. PE showed a strong negative correlation with IC (−0.79), indicating that as IC increases, PE significantly decreases. PE and AVID exhibited a strong positive correlation (0.866), i.e., a positive interactive relationship. IC and AVID displayed a moderately strong negative correlation (−0.692), where higher IC is associated with lower AVID. This may suggest that enhanced AVID could help mitigate the perception of IC.

Variable discriminant validity: Using 0.85 as the threshold for discriminant validity, correlations below this value indicate a clear distinction between variables. IC’s correlations with PE (−0.79) and AVID (−0.692) suggest good variable discrimination. PE’s correlations with AVID (0.866) and PEOU (0.854) indicate a high degree of relatedness while maintaining distinct characteristics. Additionally, VIF tests showed no multicollinearity, further confirming that the variables exhibited strong discriminant validity.

Potential interactive effects among independent variables: Given IC’s significant negative correlations with the other independent variables, a structural model (right-side model in Figure 2) was used to explore potential interactions in real-world cognitive environments. This analysis focused on evaluating the value and application of each variable in designing age-friendly information animations.

Structural model analysis: When all three independent variables were simultaneously entered into the model, the path coefficients and explanatory effects for PEOU notably decreased. PE’s path coefficient dropped from 0.853 to 0.392, shifting from a strong to a weak effect while still maintaining a significant positive influence. Similarly, IC and AVID saw reductions in both coefficient values and effect magnitude, with weakened statistical significance yet preserving original directional impacts. These changes suggest that in real-world cognitive contexts, these variables are not simply additive, but rather interact in complex ways. In multivariable scenarios, each variable’s independent explanatory power for PEOU is subject to suppression effects.

#### 4.3.5 Questionnaire survey results

This study extracted high-quality data from 90 completed questionnaires. Rigorous statistical testing, including normality assessment, correlation analysis, and multicollinearity detection, confirmed the data distribution and significant relationships between indicators. The analysis included 27 observation indicators across four predefined variables, with data that were relatively concentrated and exhibited reasonable fluctuations.

A comprehensive assessment of the four predefined variables (PE, IC, AVID, and PEOU) revealed that the 27 indicators exhibited strong explanatory power therefor. The variables demonstrated excellent internal consistency, with factor loading coefficients >0.7 and R^2^ values (communalities) > 0.4. The measurement model displayed exceptional information-capturing capabilities and structural validity, providing a solid foundation for path analysis.

Key path effect analysis revealed that PE and AVID had strong positive relationships with PEOU, whereas IC showed a strong negative relationship with PEOU. All critical paths had f^2^ values exceeding 0.35, indicating strong relationships. Therefore, H1–H3 were confirmed.

The analysis of variable interactions revealed a significant strong negative correlation between PE and IC, a significant strong positive correlation between PE and AVID, and a moderately strong negative correlation between IC and AVID.

Compared to the “independent factor detection” results, in a model with multiple independent variables, the path coefficients and explanatory power for perceived usability notably decreased, although significant relationships remained.

The questionnaire results validated H1–H3, confirming that PE and AVID enhance PEOU and that reducing IC significantly improves audience perceptions of ease of use in different models. The findings also clarified the framework for age-friendly information animation design strategies. The study uncovered complex interactions among variables associated with elderly audiences’ perception of information animations, suggesting potential mutual interference when multiple independent variables act simultaneously.

## 5 Results and discussion

### 5.1 Independent variables and age-friendly design strategies

Through empirical methods, including interviews and questionnaire surveys, this study clarified the multidimensional mechanism behind elderly audiences’ perception of information animations, as outlined by the theoretical model based on cognitive aging theory and cognitive load theory. Specifically, improvements in PE, optimization of AVID, and reduction of IC can enhance cognitive experiences for older adults.

#### 5.1.1 Strategies for enhancing PE

PE is a critical factor influencing cognitive experience in older adults, grounded in perceptual decline theory and inhibition function theory. Research data show that PE has significant positive correlations with PEOU in both independent and joint-factor models, although the strength of the correlation varies.

Drawing on Stuart-Hamilton’s (2012) perspective on age-friendly design, which emphasizes enhancing perceptual stimulation, and incorporating Norman and Bobrow’s perceptual load theory, this study argues that information animation design for the elderly should strike a balance between stimulating perception and ensuring a comfortable PE. The specific research approach requires theoretical analysis and careful observation of elderly audiences’ cognitive needs and preferences. The primary measurement indicators for PE should include visual clarity, perceptual comfort, and adaptability to dynamic rhythms. Researchers must continually assess whether improvements in PE significantly increase IC, to find a balance between these two opposing factors.

#### 5.1.2 Strategies for reducing ICs

IC is a core factor influencing cognitive experience in the elderly. Both interview and questionnaire data consistently demonstrate that reducing IC is essential for improving the perception of ease of use. In our independent factor model, IC showed a strong negative correlation with PEOU—lower complexity led to a higher perception of ease of use. Although IC may be influenced by other variables in the joint factor model, resulting in a weaker relationship, it remains a key negatively correlated variable. IC could serve as a primary factor in inter-variable interference, and its underlying mechanism should be investigated further.

IC influences cognitive processing through metrics such as information volume, narrative structure, and semantic understanding, which are closely linked to resources allocation and working memory capacity. In information visualization environments, complexity spans multiple dimensions, corresponding to the three types of cognitive load outlined in cognitive load theory: intrinsic IC perception ↔ intrinsic cognitive load; extraneous IC perception ↔ extraneous cognitive load; and germane IC perception ↔ germane cognitive load. Intrinsic IC, which includes cognitive tasks, information content, and volume, is relatively inherent. Reducing intrinsic complexity can be achieved through information segmentation or content simplification. Extraneous IC is primarily driven by inappropriate design factors, such as redundant information (e.g., superfluous visuals, irrelevant details, or overly complex narrative structures) and mismatched audiovisual relationships. These factors are crucial in age-friendly design research, where optimization strategies can significantly improve the perception of ease of use. Germane IC pertains to cognitive processes (e.g., attention, information processing, and memory) and is largely influenced by how well the visual representation aligns with the audience’s mental models. Designing visual stimuli, narrative structures, and memory cues based on elderly audiences’ mental models can enhance cognitive efficiency, resulting in a more favorable cognitive experience.

#### 5.1.3 Optimization strategies for AVI

AVID is a key factor influencing cognitive experience in the elderly, based on perceptual decline theory, resource limitation theory, and the sensory compensation mechanism. Our interview and questionnaire data showed that AVID had a strong positive correlation with PEOU in the independent factor model, indicating that improving AVID can significantly enhance the perception of ease of use. Although AVID may be influenced by other factors in the joint factor model, it remains a key determinant.

Audiovisual information integration strategies primarily focus on two aspects: One is the audiovisual compensation effects and audiovisual information integration effects. Age-friendly audiovisual compensation design involves offering partially “redundant” descriptions of auditory and visual content to support visual information comprehension through language. The other approach activates language working memory via visual stimulation, emphasizing the role of visual stimuli in processing language information (Ware, 2022).

Audiovisual information integration design aims to enhance information processing efficiency and expand working memory capacity. However, multimodal information integration can place significant cognitive demands on the elderly (Stuart-Hamilton, 2012). Therefore, the integration approach must strike a balance, either by reducing visual complexity and information volume or by simplifying narrative sentences to make them more accessible and easier to understand.

In summary, the three predetermined independent variables and validated hypotheses together form a comprehensive theoretical model and an age-friendly information animation design strategy framework, highlighting the multidimensional factors influencing PEOU for elderly audiences: enhance perceptual stimulation and experience to foster elderly audiences’ attention and cognitive engagement with dynamic visual information; reduce IC to minimize cognitive resource use and improve information processing efficiency; and improve audiovisual information integration to enhance information attention and memory retention.

### 5.2 Dynamic balance of multifactor relationships and design strategy implementation

Given the potential complex interactions among the three identified independent variables, implementing an age-friendly design strategy requires a holistic approach that maintains a dynamic balance across these variables.

When implementing strategies to reduce IC, enhance PE, and integrate audiovisual information simultaneously, a simple cumulative approach may be ineffective. Instead, a mutually constraining and dynamically adjustable system is required. For example, enhancing perceptual stimulation and experience could increase IC, necessitating simultaneous control of overall complexity during the design process.

Given the inverse relationships between IC and the other two variables, enhancing audience PE and AVID requires establishing a “complexity threshold” control mechanism. When improvements in a single dimension risk creating excessive complexity, immediate simplification or compensatory strategies should be applied. This includes controlling the critical thresholds for PE enhancement and ensuring AVI does not overly tax cognitive resources. Conversely, reducing complexity must not compromise the audience’s PE or the integration of information.

Any adjustment in a single dimension requires a synchronized assessment of its potential impacts on the other two dimensions. The goal is to enhance the cognitive experience for elderly audiences while avoiding isolated or static design approaches.

### 5.3 Cognitive experience scale for dynamic visual information targeting elderly audiences

In addition to exploring age-friendly design strategies for information animation, this research developed and applied a scale to measure the cognitive experience of dynamic visual information for elderly audiences. The scale was created through theoretical exploration, multigroup in-depth interviews, case studies, questionnaire surveys, and data analysis. It primarily includes four dimensions: PE, IC, AVID, and PEOU.

Through multidimensional and structural validity verification, all observational indicators in the scale demonstrated high internal consistency and validity, ensuring scientific rigor and reliability in measuring key variables of elderly cognitive experience with dynamic visual information. Correlation and path analyses between variables further confirmed the scale’s ability to consistently capture the impact of dynamic visual information on elderly cognitive load and experience.

The scale’s indicator selection is grounded in cognitive aging theory, cognitive experience theory, dynamic visual design theory, and age-friendly design practical experience, ensuring high universality. The sample encompassed elderly populations varying in age and general cognitive level, with validation results demonstrating high external validity and confirming the scale’s reproducibility and practical application value.

## 6 Conclusion

Grounded in cognitive aging theory and cognitive load theory, and incorporating insights from in-depth interviews with elderly audiences and design experts, the research identified three core variables influencing elderly audiences’ cognitive experience with information animation: PE, IC, and AVID.

The research began with a literature review and in-depth interviews to understand the cognitive needs and preferences of elderly audiences. A questionnaire was then designed with 27 observational indicators across four dimensions. A heat stroke knowledge animation was selected as the research case, and 90 elderly participants were invited to participate. Quantitative data analysis was performed to validate the relationships between variables and their impact pathways on PEOU.

Factor analysis of the questionnaire data independently validated three hypotheses: both PE and AVID positively influence PEOU, while reducing IC significantly enhances PEOU.

Compared to the independent factor analysis results, the path coefficients and explanatory effects of the variables on PEOU significantly diminished in the multivariate interaction model. This highlights the complex interactive mechanisms among the independent variables in relation to elderly audiences’ perception of information animations, where variables may potentially interfere with each other.

However, when multiple factors interacted, the influencing paths of the three independent variables remained significantly positive or negative, indicating that PE, IC, and AVID are core cognitive factors. These variables exhibited relatively stable and intrinsically linked characteristics in relation to elderly audiences’ information reception process.

Through questionnaire data analysis, this study clarified the multidimensional mechanisms of elderly audiences’ information animation perception, as revealed by the theoretical model based on cognitive aging theory and cognitive load theory. It identified key age-friendly information animation design strategies: enhancing PE, optimizing AVI, and reducing IC. These strategies could improve the age-friendly design framework for elderly information reception.

Simultaneously, this research suggests that cognitive influencing factors are not independent, but exhibit subtle interactions and dynamic balance. Age-friendly information animation design should not simplify the linear impacts of individual variables but rather consider the combined effects of multiple factors. Interactions between variables may lead to non-linear changes in the original relationships, requiring designers to adopt a more cautious and flexible approach. Future research should further explore the complex mediating or moderating mechanisms of these variables to gain a deeper understanding of how elderly audiences perceive information animations.

This research developed and applied a scale to measure the cognitive experience of dynamic visual information for elderly audiences. The scale includes four primary dimensions: PE, IC, AVID, and PEOU. The selection of indicators was grounded in cognitive aging theory, cognitive experience theory, dynamic visual design theory, and practical experience in age-friendly design, ensuring strong universality. The sample comprised elderly populations varying in age and cognitive level. The validation results demonstrated high external validity, with the scale exhibiting high replicability and practical application value.

The research findings provide theoretical foundations and point to design thinking pathways for age-friendly dynamic visual information design. Key innovations of the research include:

Offering insight into evidence-based age-friendly design strategies framework. The framework not only establishes independent influence paths of variables on PEOU but also reveals the interactive relationships among variables. It provides multidimensional theoretical guidance for design practices, serving as a theoretical foundation and evaluation criterion for decision-making in animated information design.

Uncovering the multidimensional complexity of elderly cognitive processing of dynamic visual information. The empirical study clarifies the complex interaction mechanisms among influencing factors, emphasizing the dynamic balance required in the implementation of design strategies.

Developing a scale applicable to research on age-friendly information animation design. A measurement tool was developed, encompassing four dimensions: PE, IC, AVID, and PEOU. The scale underwent rigorous reliability and validity testing, ensuring the reliability and effectiveness of the measurement results. It provides specific observational indicators, operationalizing abstract theoretical constructs, and can be applied to subsequent related research, demonstrating strong generalizability and practical utility.

## 7 Limitations and future research

The theoretical model for age-friendly information animation design strategies was constructed based on elderly cognitive theory and cognitive load theory. The study focused only on three core independent variables directly related to media design-PE, IC, and AVID (controllable factors in design practice)-and did not address all dimensions of elderly people’s information animation perception. The analytical framework could be expanded to include age-related variations, individual cognitive level, prior knowledge, mental models, and representational processes. The preliminary data revealed the intricate interplay between variables, suggesting that the existing joint factor model provides a foundational understanding, yet may be unable to fully capture elderly cognitive mechanisms. The observed complexity of the variable interactions points to the need for more sophisticated analytical approaches to better understand the nuanced cognitive processes of elderly audiences. Subsequent research could expand the theoretical model to incorporate additional cognitive dimensions, develop more sophisticated measurement instruments, and explore more comprehensive interaction mechanisms between variables.

Additionally, specific methods from this strategic framework should be applied to optimize age-friendly animation cases and conduct comparative testing. This will enable the exploration of dynamic implementation pathways and the evaluation of design strategy effectiveness, ultimately leading to the development of concrete and actionable age-friendly design guidelines.

By clearly outlining these research boundaries, we aim to stimulate further academic inquiry and deepen our understanding of age-friendly information design. This study serves as a critical starting point, encouraging researchers to build upon and refine our preliminary insights.

## Data Availability

The original contributions presented in the study are included in the article/supplementary material, further inquiries can be directed to the corresponding author/s.

## Author’s note

Xuan Yang is DFA and Professor at the College of Fine Arts in Guangdong Polytechnic Normal University, China. Current research interests is visual design thinking and dynamic visual design.

Feng Gan is a Ph.D. and Professor at the School of Art in Southeast University, China. He is dean of Culture and Tourism Industry Think Tank Chinese Art Evaluation Institute. Current research interests is art theory research.
